# Improving the knowledge and adherence of nursing staff to infection control ecommendations: a quality improvement pilot program

**DOI:** 10.1186/2047-2994-4-S1-P272

**Published:** 2015-06-16

**Authors:** V Sauvan, C Hudry, Y Registe-Rameau, B Huttner, D Pittet

**Affiliations:** 1 Infection Control Unit, HUG, Geneva, Switzerland; 2 Div. Internal Med&Rehab, HUG, Geneva, Switzerland

## Introduction

Since 2003, all nursing staff at HUG follow a mandatory course in infection control in the context of an institutional program. As part of a quality improvement pilot program we implemented a knowledge assessment tool in 2 units of a 300-bed geriatric hospital.

## Objectives

We aim to assess the nursing staff’s theoretical knowledge of infection control and its application in everyday practice.

## Methods

During the first period (01-04/2014) all nurses and nursing assistants in the 2 participating units were assessed for their theoretical knowledge of infection control during a 30-minute interview using a predefined questionnaire with 12 items. During the same period adherence to hand hygiene was measured based on the WHO framework. During a second period (4-6/2014) individual and group-level feedback about knowledge scores and hand hygiene adherence was provided. Individuals with suboptimal performance in either domain were targeted for individual training sessions. During a third period (6-12/2014) hand hygiene compliance and the implementation of infection control measures was audited.

## Results

21 nurses and 13 nursing assistants were assessed during the 1st period. None remembered to have received training in infection control. 5 caregivers reached the maximum knowledge score and had hand hygiene adherence > 80%. 5 caregivers reached the maximum knowledge score but had a hand hygiene compliance < 50%. 1 caregiver reached low scores both with regard to knowledge and hand hygiene compliance. During the 3rd period overall hand hygiene compliance of 6 randomly selected caregivers having participated in the 1^st^ phase increased from 59 to 98 %. 14 infection control measures were audited and all fulfilled the predefined criteria for adequacy.

## Conclusion

Despite a long institutional culture of patient safety and infection control only 15% of the nursing staff had very good theoretical knowledge of infection control and were able to implement this knowledge into good adherence to hand hygiene. This quality improvement pilot program made the whole team reflect on their practices and made it possible to identify caregivers in need of individualized training.

## Disclosure of interest

None declared.

